# Supplementary material for: Factors associated with malaria in indigenous populations: A retrospective study from 2007 to 2016
Source: PLoS One. 2020 Oct 21;15(10):e0240741. doi: 10.1371/journal.pone.0240741 (PMC7577477; doi:10.1371/journal.pone.0240741)
Supplement: S1 Table — Amazonas state, Brazil, 2007 to 2016. (DOCX) [file pone.0240741.s001.docx]

S1 Table. Distribution of cases of malaria by municipality. Amazonas state, Brazil, 2007 to 2016

|  | Indigenous* | | Non-Indigenous** | | Missing | |
| --- | --- | --- | --- | --- | --- | --- |
| MUNICIPALITY | n | % | n | % | n | % |
| Alvarães | 1174 | 0.75 | 8625 | 3.41 | 8794 | 1.81 |
| Amaturá | 492 | 0.32 | 108 | 0.04 | 223 | 0.05 |
| Anamã | 21 | 0.01 | 45 | 0.02 | 364 | 0.07 |
| Anori | 1 | 0.00 | 25 | 0.01 | 248 | 0.05 |
| Apuí | 46 | 0.03 | 548 | 0.22 | 3175 | 0.65 |
| Atalaia do Norte | 21475 | 13.78 | 7747 | 3.06 | 5801 | 1.19 |
| Autazes | 285 | 0.18 | 829 | 0.33 | 8966 | 1.84 |
| Barcelos | 19426 | 12.46 | 8177 | 3.24 | 9573 | 1.97 |
| Barreirinha | 2 | 0.00 | 18 | 0.01 | 30 | 0.01 |
| Benjamin Constant | 3866 | 2.48 | 5387 | 2.13 | 4767 | 0.98 |
| Beruri | 311 | 0.20 | 336 | 0.13 | 2372 | 0.49 |
| Boa Vista do Ramos | 0 | 0.00 | 30 | 0.01 | 41 | 0.01 |
| Boca do Acre | 90 | 0.06 | 1155 | 0.46 | 3631 | 0.75 |
| Borba | 2748 | 1.76 | 327 | 0.13 | 16614 | 3.41 |
| Caapiranga | 2 | 0.00 | 124 | 0.05 | 483 | 0.10 |
| Canutama | 75 | 0.05 | 916 | 0.36 | 279 | 0.06 |
| Carauari | 673 | 0.43 | 6265 | 2.48 | 3182 | 0.65 |
| Careiro | 11 | 0.01 | 1918 | 0.76 | 15385 | 3.16 |
| Careiro da Várzea | 285 | 0.18 | 1034 | 0.41 | 1482 | 0.30 |
| Coari | 699 | 0.45 | 11732 | 4.64 | 33164 | 6.81 |
| Codajás | 0 | 0.00 | 439 | 0.17 | 1077 | 0.22 |
| Eirunepé | 4856 | 3.12 | 28509 | 11.28 | 12085 | 2.48 |
| Envira | 576 | 0.37 | 1145 | 0.45 | 142 | 0.03 |
| Fonte Boa | 160 | 0.10 | 839 | 0.33 | 739 | 0.15 |
| Guajará | 41 | 0.03 | 9933 | 3.93 | 7796 | 1.60 |
| Humaitá | 1458 | 0.94 | 483 | 0.19 | 11064 | 2.27 |
| Ipixuna | 3631 | 2.33 | 22136 | 8.76 | 7834 | 1.61 |
| Iranduba | 20 | 0.01 | 4929 | 1.95 | 7523 | 1.55 |
| Itacoatiara | 68 | 0.04 | 700 | 0.28 | 12844 | 2.64 |
| Itamarati | 365 | 0.23 | 4367 | 1.73 | 146 | 0.03 |
| Itapiranga | 0 | 0.00 | 84 | 0.03 | 988 | 0.20 |
| Japurá | 1706 | 1.09 | 136 | 0.05 | 1753 | 0.36 |
| Juruá | 1415 | 0.91 | 2046 | 0.81 | 2264 | 0.47 |
| Jutaí | 3401 | 2.18 | 5053 | 2.00 | 5449 | 1.12 |
| Lábrea | 9113 | 5.85 | 19761 | 7.82 | 7857 | 1.61 |
| Manacapuru | 152 | 0.10 | 1677 | 0.66 | 1308 | 0.27 |
| Manaquiri | 113 | 0.07 | 384 | 0.15 | 123 | 0.03 |
| Manaus | 840 | 0.54 | 41746 | 16.52 | 134594 | 27.65 |
| Manicoré | 965 | 0.62 | 1289 | 0.51 | 15979 | 3.28 |
| Maraã | 1353 | 0.87 | 2331 | 0.92 | 2118 | 0.44 |
| Maués | 2338 | 1.50 | 478 | 0.19 | 832 | 0.17 |
| Nhamundá | 2 | 0.00 | 13 | 0.01 | 1083 | 0.22 |
| Nova Olinda do Norte | 32 | 0.02 | 151 | 0.06 | 1295 | 0.27 |
| Novo Airão | 186 | 0.12 | 352 | 0.14 | 3316 | 0.68 |
| Novo Aripuanã | 607 | 0.39 | 862 | 0.34 | 5884 | 1.21 |
| Parintins | 15 | 0.01 | 66 | 0.03 | 462 | 0.09 |
| Pauini | 1465 | 0.94 | 4553 | 1.80 | 2504 | 0.51 |
| Presidente Figueiredo | 15 | 0.01 | 4241 | 1.68 | 9127 | 1.88 |
| Rio Preto da Eva | 60 | 0.04 | 2488 | 0.98 | 8227 | 1.69 |
| Santa Isabel do Rio Negro | 5878 | 3.77 | 156 | 0.06 | 11277 | 2.32 |
| Santo Antônio do Içá | 1144 | 0.73 | 7342 | 2.90 | 5574 | 1.15 |
| São Gabriel da Cachoeira | 32943 | 21.13 | 591 | 0.23 | 30159 | 6.20 |
| São Paulo de Olivença | 12709 | 8.15 | 168 | 0.07 | 5801 | 1.19 |
| São Sebastião do Uatumã | 0 | 0.00 | 406 | 0.16 | 2399 | 0.49 |
| Silves | 1 | 0.00 | 32 | 0.01 | 1422 | 0.29 |
| Tabatinga | 10258 | 6.58 | 2418 | 0.96 | 9945 | 2.04 |
| Tapauá | 3961 | 2.54 | 4863 | 1.92 | 1211 | 0.25 |
| Tefé | 1732 | 1.11 | 13471 | 5.33 | 25325 | 5.20 |
| Tonantins | 37 | 0.02 | 121 | 0.05 | 527 | 0.11 |
| Uarini | 549 | 0.35 | 6326 | 2.50 | 6856 | 1.41 |
| Urucará | 29 | 0.02 | 309 | 0.12 | 1216 | 0.25 |
| Urucurituba | 0 | 0.00 | 18 | 0.01 | 42 | 0.01 |
| Total | 168830 | 100.00 | 92056 | 100.00 | 560079 | 100.00 |

Source: Epidemiological Surveillance Information System for Malaria, data obtained in January, 2018.

*Indigenous is the combination of an individual with a declared indigenous status or having the indigenous village as a place of infection.

**Non-indigenous is the combination of all other races, excluding the indigenous and missing data race.
