# Supplementary material for: Factors associated with malaria in indigenous populations: A retrospective study from 2007 to 2016
Source: PLoS One. 2020 Oct 21;15(10):e0240741. doi: 10.1371/journal.pone.0240741 (PMC7577477; doi:10.1371/journal.pone.0240741)
Supplement: Attachment — Submitted filename: Review_14092020.pdf [file pone.0240741.s003.pdf]

The aim of this study was to identify factors associated with malaria in indigenous people, in Amazonas state, Brazil. The study used secondary data obtained from the Brazilian malaria surveillance program.
Some one minor detail that require correction:

Introduction

- Please correct the last sentence: "The aim of this study was to The aim of this study was to..."
