# Supplementary material for: Factors associated with malaria in indigenous populations: A retrospective study from 2007 to 2016
Source: PLoS One. 2020 Oct 21;15(10):e0240741. doi: 10.1371/journal.pone.0240741 (PMC7577477; doi:10.1371/journal.pone.0240741)
Supplement: Attachment — Submitted filename: Response to Reviewers_PONE-D-20-05382.docx [file pone.0240741.s002.docx]

Dr. Maria Jacirema Ferreira Gonçalves	phone ++ 55 (92) 99624-8192

Public Health Researcher

To The Academic Editor Dr. José Moreira

PLoS ONE

**PONE-D-20-05382 “Factors associated with malaria in indigenous populations: a retrospective study from 2007 to 2016”**

We would like to re-submit the manuscript above in which we considered the editor and reviewer's comments.

We send attached the manuscript with track changes.

We very much hope you will decide to publish the paper. Please do not hesitate to contact us should you require any additional information.

Yours sincerely,

The Authors

Dr. José Moreira

Academic Editor, PLOS ONE

Manaus, August 21, 2020

Dear Editor,

Initially, we would like to thank yours and the reviewer's attention in evaluating our manuscript entitled **"Factors associated with malaria in indigenous populations: a retrospective study from 2007 to 2016** " code: **PONE-D-20-05382**.

We consider relevant all the comments of the editor and reviewers. In order to improve our manuscript and comply with PlosOne recommendations, we developed a thorough review and accepted almost all of the suggestions.

As stated that the article does not fully meet PLOS ONE’s publication criteria, we present below a reply to each comment pointed out by reviewers. We also send attached a completely redesigned version, including changes using track changes in our manuscript for evaluation.

**Editor's comments:**

General:

Please rewrite the abstract as suggested by one of the reviewers

Perform language copyediting for the entire text:
Authors’ reply: We rewrote the abstract and performed a language review in all the manuscript.

Methods

Provide sufficient details about the SIVEP, SIVEP-Malaria, and MIS. Similarly, contextualize the readers your study setting, specifically the nuances about the Amazon state, the tribal villages, the national malaria program guidelines. How community healthcare workers perform the diagnosis of malaria in indigenous people? Any surveillance underway?

How did you handle missing data?

Authors’ reply: In order to address the actual understanding of our information system, malaria surveillance and indigenous area, we explain these aspects in detail in the method.

About missing data, we present in the results the percentage of missing data for all variables. In the Table 1 all the data are shown, including a column of race missing data. In the method we describe the imputation of indigenous race, considering an indigenous person someone who had the village as the place of probable infection and who did not have a race registration.

Results

Does the outcome and severity of malaria infection vary according to race? Could you present malaria incidence by year? Is there any variation of malaria incidence in indigenous people throughout the study period?.

Authors’ reply: We consider using the malaria incidence indicator with caution, as the numerator can be repeated, since a person can have more than one episode of malaria per year. In general, there is an annual parasitic incidence (positive cases per population times 1000). In the case of stratification by race, we have a problem in the numerator due to flaws in the registration of race in the information system (that is why we performed this analysis in an attempt to imput data), and in the denominator, as the indigenous population is not geographically distributed as the general population, obeying geographical limits. In addition, it is a population that, due to its way of life, presents population dynamics (migration, deaths and births) that needs to be counted in loco. With secondary data, in a large series of data like this, we cannot reconstruct that data. Another aspect is that we only have demographic census data for 2010, and we would have to use projection strategies and population estimates, with methodologies applicable to indigenous populations.

- Discussion

Address the limitations of your findings considering the drawbacks of working with national epidemiological information systems.

Authors’ reply: We describe our potential limitations and considerations about using national epidemiological information systems. We also combined your request with other reviewers.

**- Journal Requirements:**

. The manuscript has images which may be copyrighted

Authors’ reply: We decided to exclude Figure 1. This decision occurs because we could not provide an explicit written permission to publish Figure 1 and considering its elimination does not implicate in the adequate understanding of our manuscript.

Reviewers' comments:

Reviewer's Responses to Questions”

1. Is the manuscript technically sound, and do the data support the conclusions?

Authors’ reply: As there was two reviewers that attributed it "partially", the authors reviewed the entire article in order to support our conclusions based on the data presented.

2. Has the statistical analysis been performed appropriately and rigorously?

Authors’ reply: The authors would like to thank the positive evaluation regarding the adequate statistical analysis of the manuscript

3. Have the authors made all data underlying the findings in their manuscript fully available?
Authors’ reply: Due to our laws, we cannot make the data used freely available. However, we declare the availability of the data, upon request to the government department, responsible for the malaria data. This information was filled in the submission system metadata.

4. Is the manuscript presented in an intelligible fashion and written in standard English?

Authors’ reply: We conducted a complete review of the English language. If it is necessary, we can provide a new review after all modifications.

**Reviewer #1**: The authors chose an interesting subject, with few approaches on literature regarding indigenous malaria in Brazil, but needs some revision concerned the following aspects:

1) Developing the indigenous lifestyle that contributed to presented results
Authors’ reply: We present this information supported by literature, since our analysis used secondary data, we could not conduct field observation. In combination with the comments of all reviewers, these considerations have been incorporated throughout the text

2) In Methodology the paragraph about including as indigenous people those which referred living in "aldeias" it was not clear the meaning of the sentence " The only possible cases of erroneous inclusion would
be the small number of health professionals who access such places to provide indigenous
health care. The authors should explain what they had on mind with this assumption.
Authors’ reply: The idea with the imputation of race data was to ensure that we would not be inflating indigenous race information. Therefore, we affirm that the option of considering "village" as a place of probable infection, and thus classifying the case as indigenous would lead to few classification errors, since the non-indigenous people who enter the villages are the health professionals, who are in very low numbers and would be more likely to be a case of malaria, the professional would fill in the information system with his race and would not be a case of missing data.

Considering that it was not very clear in the text, we changed the wording.

3) In Methodology - Definition of active and passive case malaria surveillance should be provided in methodology
Authors’ reply: We included the definition of active and passive case malaria surveillance in the method.

4) In Methodology - It should be well defined how many thick blood smears were considered to establish malaria diagnosis since "the number of parasites in thick smears was graded using the plus system scale:"
Authors’ reply: parasite per 100 thick film fields. This number is included in the text.

5) In Methodology - please provide the number and the data of the approval of the study by the Ethics Review Board of the Federal University of Amazonas.
Authors’ reply: we added the information.

6) The authors should provide percentages of some observed variables such as age, parasitemia to better charactherize their found results.
Authors’ reply: There are reviewers who recommend not repeating the numbers inside the tables, as it would be a duplicate of information. As the focus of the analysis is the associated factors, we present these data numerically, in addition to those already available in the table.

7) Necessity of reviewing table 1 concerning some variables that do not match the mentioned percentage on parenthesis; the absolute values of some variables do not reach 100%, being low or above this percentage.
Authors’ reply: The percentage is calculated in the column. We added this information in the table note

8) Please, add in Fig 1 the percentages observed, instead of informing only colors in the polygons -dark and light blue, respectively to low and high percentages
Authors’ reply: Considering other comments, Fig 1 was removed.

9) To strength the discussion (and even the conclusion) about the biggest obstacles to malaria treatment in indigenous populations that is getting access to them due to their geographical location as well as their dispersion in the state of Amazon, authors should include a map with this information on the results
Authors’ reply: We could not provide a map. We remark the cited reference in this paragraph has a map that can help to understand.

10) Based on the results, two topics in the conclusion need to be better addressed in the discussion - the reason why indigenous peoples of the state of the Amazon is "obviously" neglected and the significant predominance of passive case malaria diagnosis.
Authors’ reply: this aspect is addressed, together with comments of the others reviewers.

We discuss that one possible explanation is that people had many episodes of malaria during their live. So when they have symptoms, they already suspect of malaria, and look for diagnosis.

**Reviewer #2**: The authors present malaria burden in the Amazonas in the last decade and identify the factors associated with it. However, the authors need to address certain points before the manuscript is suitable for publication.

Page 4

1. Data of cross-sectional studies needs further explanations (how data was collected, target population, ACD vs PCD...). This is a key aspect that requires visibility in the manuscript.

Authors’ reply: We added explanation about active and passive case surveillance in the method.

2. A paragraph explaining a socio-economic description of the state of Amazonas (race distribution, wealth), and the epidemiology of malaria in the state is missing (access to malaria prevention, first-line treatment, vivax vs falciparum, ...).

Authors’ reply: These information are now provided in the study site sub-section.

Page 5:

3. Which diagnostic test was used for confirmation of malaria? Was it performed at health facility or actively in the community? If only microscopy was used it must be stated as limitation in the paragraph were low RDT usage is discussed.

Authors’ reply: We try to address these issues in the text. we hope to meet your expectations.

4. Expand the multiple imputation methods in an appendix.

Authors’ reply: We did not performed multiple imputation. According to all the reviewers suggestions, we try to better explain in the method.

Page 6:

5. Variables included: what about malaria prevention (ITNs and IRS), and women being pregnant? Do the authors think that missing to include these variables might have an impact on the results?

Authors’ reply: The problem with analysis with secondary data is the absence of variables that can clarify some aspects. As we only work with confirmed positive cases, we do not have data from the entire population to compare preventive measures. On the other hand, as indigenous people sleep in hammocks, the effectiveness of using ITN is reduced. As for the data of pregnant women, we believe that it does not impact the results found, and perhaps it deserves a specific analysis with primary data..

6. I understand that job occupation and wealth (or socio-economic status) were not collected. If not available, they need to be included in study limitations in the discussion.

Authors’ reply: Thank you for suggestion. We added in the limitations.

Page 7:

7. It is briefly discussed at the end of the manuscript, but did the authors address the issue of malaria relapses associated with P. vivax? If done, how were relapses excluded/differentiated?

Authors’ reply: We did not deepen our analysis on the cases of relapses, because as a new case, it certainly entered the database and had its factor associated with malaria measured. The data have already been obtained without identifying the subjects, which does not allow an adequate assessment of cases of relapse..

Page 8:

8. It is important that the authors clarify the terms active case detection and passive case detection in the methods, and include further interpretations in the discussion.

Authors’ reply: We added this information and pooled with all reviewers comments .

Page 12:

9. The first two paragraphs can be combined to avoid repetition.

Authors’ reply: Yes, it was rewritten emphasizing the adjusted analysis result.

Discussion section:

10. This section needs a limitation paragraph and address potential biases of the study.

Authors’ reply: We combined recommendation of all reviewers and present the study limitations.

**Reviewer #3**: The aim of this study was to identify factors associated with malaria in indigenous people, in Amazonas state, Brazil. The study used secondary data obtained from the Brazilian malaria surveillance program.

Some major comments and minor details that require further information or consideration:

Abstract

- There is no sentence about background, the first phrase is the objective without basic information of where, when, why.

- The period is repeated in Methods and Results.

- The study design is not described.

- There is no information about both data systems are Brazilian systems.

- The basic statistical analysis is not too important to describe in the abstract. Definitions or study local details or variables is more important than those.

- The phrase: “This state has the highest proportion of indigenous peoples and malaria in Brazil” in the Results section is a result of the study or an information about the study site? If it is an information should be in the Method section or in Background.

- There is no results about the factors cited as associated malaria and indigenous people (measures of association, 95% IC, p).

- The conclusion is not in accordance with the results presented in the abstract.

Authors’ reply: The authors present a new abstract version. With respect to the numerical values (measures of association, 95% IC, p) of the results, according to other reviewers, it would not be appropriate to present it in the abstract.

Introduction

- There is no dot in the end of the second sentence.

- In the sentence: “The production and transmission of malaria”, the “production of malaria” could be replaced by development or occurrence.

- Page 4: last sentence: The sentence would better written as: The aim of this study was to identify factors associated with malaria in indigenous people.

Authors’ reply: We accepted all suggestions.

Methods

- The order of the sub-sections could be changed to better understand the methodology. First of all, it is necessary to describe the study site, study population, study design and ethical considerations. After that, describe the procedures, the data analysis, the definitions of variables.

- The study design did not mention the use of secondary data.

- “Definitions” sub-section is related to “Study population”.

- The Study location could have more detailed. For instance, how many tribes/villages (aldeias), how many malaria screenings sites, amount of the indigenous population.

- The sentence “The database was organized and analyzed using Stata software version 13” could be relocated to Data analysis sub-section.

- To make sense I suggest to better describe the procedures, step by step. First, from where the data were obtained, describe the surveillance systems in Brazil, the notification case form. Then, the selection of variables and definitions.

Authors’ reply: We changed the sub-sections and their order in the methods, pooling with the suggestions of all reviewers. We also accepted all the suggestions in the method and changed the text.

Results

-Page 8: 1st paragraph: “Therefore, cases without race information were reclassified for the purpose of this study. These were classified as indigenous when they had their indigenous race registered or because they were a case where the probable place of infection was the indigenous village. Thus, 42,702 subjects, who previously had missing information on race, received an indigenous classification based on this criterion.” If they had their indigenous race registered so they were not a case without race. Those sentences should be rewritten. First describe how indigenous was identified. Then, describe what you did in the situation of cases without race information. Those sentences could be relocated to Methods section.

- Page 8: There are no results in second, third and fourth paragraphs. They are summary of results. Summary of results is better located in paragraphs of Discussion section to introduce the discussion of the results. In the Results section it is necessary write the results, reporting n as well as %, measures of association, 95% IC, p. If a descriptor is used in the sentence (e.g. "the largest percentage") include number and percentage in brackets (n=#, %).

- Page 12: the first paragraph is different from the second paragraph. In the first one there is no result described, in the second one there are for some variables.

- Page 12: second paragraph: is better to describe as variables associated with indigenous population, not as significant variables.

- Page 12: second paragraph: some variables has results in brackets, some has not. Describe all.

- Page 12: second paragraph: “parasitemia in plus system scale is higher in the last strata of the scale” does not make sense. What does represent? Describe as higher parasitemia is associated with malaria in indigenous population.

- Table 2, page 14: describe “Other” Results of the Test, in the table legend, for example.

Authors’ reply: We understand the reviewer's request for a description of the results. However, in line with other reviewers who request not to repeat the numbers presented in the tables, the authors reformulated the text combining the requests of the various reviewers.

Specifically about your comments, we transferred part of the text to the methods and described the results in order to meet your requests.

Discussion

- Page 16, last paragraph: about what results the paragraph is related? The paragraph could be relocated to Study location sub-section.

- Page 17: “After diagnosis of malaria, treatment was immediately prescribed and administered free of charge to all the patients [25].” This statement is redundant. It could be relocated to Study location sub-section, to better illustrate the Brazilian malaria program. It is important to briefly comment about indigenous malaria health program, if is apart of Brazilian general health program or not.

- Page 17: “Regarding the Plasmodium species, the present study showed similar results to those performed in non-indigenous populations, with predominance of P. vivax as an infectious agent [27, 28]. An epidemiological malaria survey found prevalence of P. vivax cases, followed by P. falciparum and mixed infection cases (P. vivax + P. falciparum) [29], a result similar to that found in our study.” About these affirmations, the results showed the prevalence of P. falciparum in indigenous population was 13.09% while the prevalence in non-indigenous was 7.92%. For mixed infection, the prevalence in indigenous population was 0.93% and non-indigenous was 0.33%. We know P. falciparum and mixed infection P.falciparum/P.vivax are different in terms of morbidity, mortality, costs etc. P. vivax is the predominant species in both population but there is a difference in the distribution of species and consequently likely in the burden of disease in the indigenous population.

- There are no considerations about limitations of the study.

- The factors identified as associated with malaria in indigenous populations (table 2) were not discussed in the Discussion section.

- The results of table 3 were not discussed in the Discussion section.

- Sometimes they describe “the state of Amazonas” and “Amazon” for the apparently the same place.

Authors’ reply: We replaced “state of Amazonas” when refer to the state, and Amazon, when referred to the region, and reviewed all the text.

We displaced the pointed paragraph to the method section.

About the prevalence of clinical forms in the indigenous people, we agree with the comments and rewrote the paragraph considering the prevalence differences.

We also added discussion about the items listed and stated the study limitations

General comments

- Add line numbers in the document helps us to point the text.

- I suggest the authors to follow the STROBE guidelines on reporting of observational studies.

- It would benefit from additional editing for English language and for redaction”

Authors’ reply: Thank you for all your suggestions. We added line numbers, reviewed the compliance our manuscript the STROBE guideline and reviewed English language.
